# A new autophagy-related nomogram and mechanism in multiple myeloma

**DOI:** 10.1016/j.gendis.2023.101120

**Published:** 2023-09-21

**Authors:** Hanying Huang, Yang Li, Ziang Zhu, Yang Liu, Weida Wang, Shuzhao Chen, Xiaoping Wu, Yun Wang, Yanzhou Chen, Huanxin Lin, Yang Liang, Lingling Shu

**Affiliations:** a State Key Laboratory of Oncology in South China, Collaborative Innovation Center for Cancer Medicine, Sun Yat-sen University Cancer Center, Guangzhou, Guangdong 510060, China; b Department of Hematologic Oncology, Sun Yat-sen University Cancer Center, Guangzhou, Guangdong 510060, China; c Department of Radiation Oncology, Sun Yat-sen University Cancer Center, Guangzhou, Guangdong 510060, China; d Key Laboratory of Neurosurgery in Guangdong Province, Southern Medical University, Guangzhou, Guangdong 510060, China; e Department of Neurosurgery, Zhujiang Hospital, Southern Medical University, Guangzhou, Guangdong 510060, China; f State Key Laboratory of Pharmaceutical Biotechnology, The University of Hong Kong, Hong Kong 999077, China

Multiple myeloma (MM) is the second most common hematological tumor. It is characterized by high drug resistance, easy recurrence, and poor prognosis, and remains incurable. Various models or scoring modalities can be used to predict the survival prognosis of MM patients; however, these predictions are still not accurate enough. We have previously found that scorings related to bone marrow microenvironment metabolism can improve predictive efficacy.^1^ Given the importance of autophagy as a stress-induced self-degradation process critical for cell survival, particularly in the hypoxic bone marrow microenvironment of MM, autophagy-related genes are considered crucial.^2^ We constructed an Autophagy Risk Score (ARS) model using 11 survival-associated autophagy-related genes (ARGs). Multivariate analysis showed that ARS was an independent predictor of survival. Most importantly, the combination of an international staging system (ISS) and the ARS model into a new nomogram model can improve the accuracy of MM survival prediction. Additionally, targeting the autophagic gene *ARNT* could potentially overcome drug resistance to bortezomib in the bone marrow microenvironment of MM. The workflow is presented in Figure S1A. The study was approved by the Ethics Committee of Sun Yat-sen University Cancer Center.

We identified 38 ARGs in the training dataset (GSE24080) by univariate Cox regression analysis, including *ARNT* gene that was significantly associated with the prognosis of MM patients (hazard ratio = 8.72, *P* = 0.019; Fig. S1B). Multivariate Cox regression analysis was used to select 11 ARGs and develop an ARS model, with an optimal weighting coefficient for each gene (Table S1 and Fig. S1C). ARS were calculated for each subject, according to the ARS formula, and subjects were then divided into high-risk and low-risk cohorts, according to the median ARS in the corresponding dataset. The sensitivity and specificity of ARS were assessed by time-ROC analysis. AUC (area under curve) value for 3-year survival was 0.72 (0.66, 0.76) in the GSE24080 training dataset (Fig. 1A). Corresponding 3-year survival AUC values were 0.65 (0.59, 0.70) for the GSE136337 validation dataset, and 0.91 (0.70, 1.00) for the GSE57317 validation dataset (Fig. S1D, E). Kaplan–Meier curves were used to compare the survival of the high-risk and low-risk groups in the training and validation data sets. In the GSE24080 training dataset, the 5-year survival rate in the high-risk group was 56%, which was dramatically lower than that of the low-risk group (81%) (Fig. 1B; *P* < 0.05). Consistently, the 5-year survival rates were also markedly lower in the high-risk groups in both validation datasets (GSE136337 and GSE57317) (Fig. S1F, G; *P* < 0.05).

**Figure 1:**
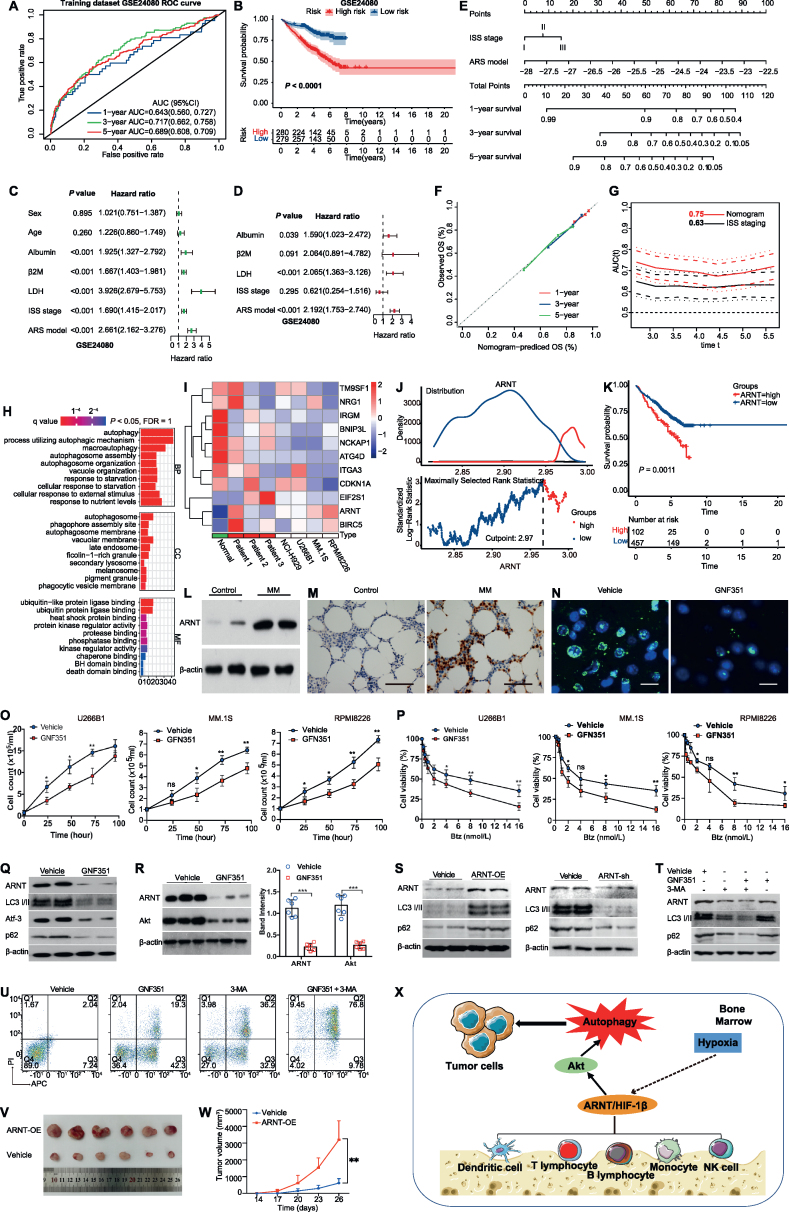
Implications of ARGs and ARNT in multiple myeloma (MM). **(A)** Time-dependent receiver operating characteristic (ROC) analysis of prognostic model prediction of 1-, 3-, and 5-year overall survival in the training dataset **(B)** Kaplan–Meier curve of the prognostic model in the training dataset. **(C, D)** Forest plot of univariate (green) and multivariate (red) Cox regression analysis in the GSE24080 training dataset. Multi-factor inclusion criteria: single factor *P* < 0.05 and reproducible factors were excluded. **(F)** Calibration curves to predict 1-, 3-, and 5-year overall survival of the nomogram in the training dataset. **(G)** Time-dependent ROC to compare the nomogram and ISS staging in the training dataset. **(H)** Significantly enriched Gene Ontology pathways in the GSE24080 training dataset and the GSE136337 validation dataset. **(I)** Relative mRNA expression of 11 autophagy-related genes by RT-qPCR. **(J)** The optimal cutoff value for *ARNT* expression level (2.97 copies) was determined by ROC curve analysis of the training dataset (GSE24080), and patients with MM were classified into high and low *ARNT* expression groups according to the cutoff value. **(K)** Kaplan–Meier survival plots according to *ARNT* expression level in the training dataset (GSE24080; *P* = 0.0011). **(L)** Western blot analysis demonstrating ARNT expression in bone mesenchymal stem cells (BMSCs) isolated from MM patients or healthy controls. **(M)** Immunohistochemistry staining of ARNT in bone marrow biopsy samples collected from MM patients or healthy control. Scale bar = 50 μm. **(N)** The MM cell line U266B1 was treated with the ARNT inhibitor GNF351 (1 μm, 24 h). ARNT expression was assessed by immunohistochemistry staining analysis (ARNT, green; DAPI, blue). Scale bar = 10 μm. **(O)** U266B1, MM.1S, and RPMI8226 cells were cultured for 4 days in the presence of GNF351 (1 μm) or PBS as vehicle control; cell number was counted every 24 h. **(P)** U266B1, MM.1S, and RPMI8226 cells were exposed to a series of concentrations of bortezomib (0.25–16 nmol/L, 24 h), followed by a CCK-8 assay to determine cell viability. **(Q)** Western blot analysis of levels of autophagy-related proteins in U266B1 cells treated with GNF351 (1 μm, 24 h). **(R)** Western blot analysis and quantification of ARNT and AKT expression in the MM cell line, U266B1, treated with ARNT inhibitor (GNF351, 1 μm) for 24 h. **(S)** Western blot analysis of levels of autophagy-related proteins in NCI–H929 cells with ARNT overexpression or ARNT knockdown. **(T)** Western blot analysis of levels of autophagy-related proteins in U266B1 cells treated with GNF351 (1 μm, 24 h), 3-MA (10 μm, 24 h), or both. **(U)** The apoptosis rate of cells treated with GNF351 (1 μm, 24 h), 3-MA (10 μm, 24 h), or both. The data were expressed as mean ± standard deviation. ^∗^*P* < 0.05, ^∗∗^*P* < 0.01; *n* = 6. **(V)** Representative images of tumor tissue acquired from each group of mice. Humanized NRG-3GS mice were euthanized, and tumor tissue was isolated and acquired. **(W)***In vivo* tumor volume was determined with vernier calipers every 3 days, and data collected on the same day were compared between groups. **(X)** Diagram of the potential mechanism by which ARNT enhances autophagy in the bone marrow microenvironment. ARNT is expressed in various immune cells, including dendritic cells, lymphocytes, monocytes, and NK cells, in the bone marrow microenvironment. ARNT has a potential relationship with hypoxia, metabolism, and immunity in the bone marrow microenvironment. In contrast, ARNT-activated AKT signaling exacerbates autophagy in myeloma cells, leading to enhanced MM cell proliferation and drug resistance. ARS, autophagy risk score; BP, Biological process; CC, cellular component; HR, hazard ratio; ISS, international staging system; MF, molecular function.

Univariate and multivariate Cox regression analysis identified ARS and other clinical and laboratory indicators related to survival in the training dataset (GSE24080; Fig. 1C, D) and in the validation dataset (GSE136337; Fig. S1H, I). Multivariate criteria include single factor *P* < 0.05 and exclude reproducible factors. Although the GSE57317 validation dataset lacked data on clinical variables, the ARS model was an independent factor related to survival with a hazard ratio = 2.19 (1.75, 2.74; *P* < 0.001) in the training dataset (GSE24080) and hazard ratio = 1.07 (1.04, 1.10; *P* < 0.001) in the validation dataset (GSE136337). A nomogram combining ISS and ARS models was constructed in the training dataset (Fig. 1E). The calibration curve was used to show the accuracy of the nomogram assessment (Fig. 1F). The nomogram increased the accuracy of MM survival prediction from the ISS stage alone (AUC = 0.63) to a higher consistency index (AUC = 0.75) (Fig. 1G). To further explore the mechanisms involved in autophagy, we used Gene Ontology pathways to identify significant differences between high- and low-risk groups in the GSE24080 training dataset (Fig. 1H). It indicated that autophagy factors exacerbate multiple autophagic and metabolic signaling pathways.

Expression of 11 autophagic genes was detected in MM patients and cell lines; *ARNT*, *BIRC5*, and *EIF2S1* were highly expressed while 8 others had a relatively low expression, consistent with the constructed autophagic model (Fig. 1I). It has been previously reported that *ARNT*, a 1q21 gene, is highly expressed in relapsed and refractory MM.^3^ The optimal ARNT expression level cutoff value was calculated to be 2.97 copies based on the training dataset ROC curve and used to categorize MM patients into high and low expression groups (Fig. 1J). MM patients with high ARNT expression had a significantly lower survival rate than those with low expression (*P* = 0.001; Fig. 1K). Expression of *ARNT* in primary bone marrow plasma cells was significantly higher in patients with MM than in healthy controls (Fig. 1L), which was further confirmed by immunohistochemistry staining analysis (Fig. 1M).

A recent study demonstrated that knockdown of *ARNT* can reverse bortezomib resistance.^4^ To evaluate ARNT's autophagic role in MM pathogenesis, we treated MM cell line U266B1 with GNF351 and found that ARNT expression was significantly reduced (Fig. 1N). GNF351 inhibited tumor cell growth in U266B1, MM.1S, and RPMI8226 cells over 75 h (Fig. 1O). Furthermore, GNF351 also enhanced bortezomib lethality in a dose-dependent manner in MM cells by checking cell viability (Fig. 1P).

To explore the potential mechanism by which ARNT exacerbates autophagy in the bone marrow microenvironment, the GENEMANIA database was used to identify a network linking ARNT with the AKT signaling pathway (Fig. S2A). After GNF351 treatment, expression levels of ARNT and autophagy-related proteins (LC3 I/II, ATF3, and SQSTM1/p62) were reduced in MM cells (Fig. 1Q), accompanied by significant attenuation of AKT expression in MM cells (Fig. 1R). To confirm the impact of ARNT on MM cell proliferation and bortezomib sensitivity, overexpression or knockdown of ARNT was carried out in the NCI–H929 cell line. Overexpression of ARNT up-regulated expression of autophagy-related proteins LC3 I/II and p62, while knockdown of ARNT exhibited adverse effects (Fig. 1S). To test whether the ARNT-mediated effect was attributed to the autophagy signaling pathway, the GNF351 and the autophagy inhibitor 3-MA were adopted *in vitro*. Treatment of NCI–H929 cells with dual inhibitors GNF351 and 3-MA caused a stronger inhibitory effect on autophagy, as evidenced by decreased protein expression levels of ARNT, LC3 I/II, and p62 (Fig. 1T). Furthermore, both GNF351 and 3-MA could promote the apoptosis of MM cells, and the combined effect was even more vigorous (Fig. 1U).

A previous study showed that ARNT promotes bortezomib resistance by enhancing the autophagy pathway, likely via modulation of the PI3K-AKT-ARNT signaling pathway.^5^ To further verify the role of *ARNT* in MM growth, NCI–H929 cells were implanted subcutaneously in NRG-3GS mice. Compared with the vehicle group, ARNT overexpression exacerbated the tumor sizes in mice (*P* < 0.01; Fig. 1V, W). ARNT was expressed in multiple immune cell types, including endothelial cells, monocytes, dendritic cells, T cells, B cells, and NK cells, as observed through analysis of The Human Protein Atlas dataset (Fig. S2B, C). The correlation of ARNT between different immune cell type markers is presented in Figure S2D. Moreover, ARNT expression was associated with endoplasmic reticulum stress in various cell lines (Fig. S2E). Furthermore, HIF-1α could be transferred to the nucleus to bind to ARNT, which enhanced HIF signaling and may be beneficial to tumor cells. These studies point to ARNT as a potential drug target in malignant tumors.

In summary, ARNT is highly expressed in MM, enhances MM cell proliferation, and promotes drug resistance. Pharmacological inhibition of ARNT can have a synergistic effect with bortezomib to inhibit MM cell proliferation. Mechanically, ARNT mediates the proliferation of myeloma cells and drug resistance through the AKT signaling pathway; in addition, ARNT, as an autophagy-related gene, has a potential relationship with hypoxia, metabolism, and immunity in the bone marrow microenvironment of MM patients (Fig. 1X).

## Ethics declaration

The clinical study was approved by the Human Ethics Committee of Sun Yat-sen University Cancer Center (No. G2022-154-01). The animal study was approved by the Institutional Animal Care and Use Committee of Sun Yat-sen University (No. L025501202206012).

## Author contributions

Hanying Huang, Yang Li, Ziang Zhu, and Yang Liu: figure preparation and original manuscript writing; Lingling Shu and Yang Liang: manuscript reviewing and editing; Shuzhao Chen and Weida Wang: software; Yanzhou Chen: methodology; Xiaoping Wu and Yun Wang: conceptualization; Lingling Shu, Yang Liang, and Huanxin Lin: research design, project administration, and funding acquisition. All authors read and agreed to the published version of the manuscript.

## Conflict of interests

The authors declare no conflict of interests.

## Funding

L-LS was supported by the Sun Yat-sen University Hundred Talents Program (China) (No. PT19200101), 10.13039/501100003453Natural Science Foundation of Guangdong Province, China (No. 2022A1515010290), Guangdong Province Science and Technology Planning Project of China (No. 2020A1414010033), and Guangzhou Science and Technology Planning Project of China (No. 202102020430). YL was supported, in part, by Sun Yat-sen University Start-up Funding (China) (No. 201603). H-XL was supported by the 10.13039/501100001809National Natural Science Foundation of China (No. 81773103) and the Natural Science Foundation of Guangdong Province, China (No. 2017A030313617).

## References

[1] Huang H.Y., Wang Y., Wang W.D. (2021). A prognostic survival model based on metabolism-related gene expression in plasma cell myeloma. Leukemia.

[2] Rankin E.B., Giaccia A.J. (2016). Hypoxic control of metastasis. Science.

[3] Schito L., Semenza G.L. (2016). Hypoxia-inducible factors: master regulators of cancer progression. Trends Cancer.

[4] Wu C., Yang T., Liu Y. (2018). ARNT/HIF-1β links high-risk 1q21 gain and microenvironmental hypoxia to drug resistance and poor prognosis in multiple myeloma. Cancer Med.

[5] Zhou D., Liu W., Liang S. (2018). Apoptin-derived peptide reverses cisplatin resistance in gastric cancer through the PI3K-AKT signaling pathway. Cancer Med.

