# Supplementary material for: A new autophagy-related nomogram and mechanism in multiple myeloma
Source: Genes Dis. 2023 Sep 21;11(5):101120. doi: 10.1016/j.gendis.2023.101120 (PMC11145194; doi:10.1016/j.gendis.2023.101120)
Supplement: Multimedia component 1 [file mmc1.docx]

**Materials and methods**

**Datasets and subject selection**

RNA matrices and clinical data from three MM datasets, GSE24080 [21], GSE136337 [22], and GSE57317 [23], were obtained from the GEO database (http://www.ncbi.nlm.nih.gov/geo/) and data normalized across the arrays. In total, 1,038 subjects with available survival and RNA data in the three datasets were included in this study. There were no duplicate samples in the dataset. Total RNA was isolated from plasma cells enriched from bone marrow aspirates by anti-CD138 immunomagnetic bead selection. The GSE24080 dataset was used as a training set to build an ARS model. The GSE136337 and GSE9782 datasets were independent validation sets used for model verification. There were sufficient subjects with clinical covariates for univariate and multivariate Cox regression analysis in the training dataset (GSE24080; N = 557) and one of the validation datasets (GSE136337; N = 416), but not in the other validation dataset (GSE57317; N = 55). ARGs (n = 232) were extracted from the Human Autophagy Database. The workflow is presented as Figure S1A.

**Construction and validation of an ARS model**

Transcript expression profiles were normalized and log_2_ transformed. Univariable Cox regression analysis was performed to identify autophagy genes correlated with prognosis (P < 0.02). A multivariate Cox regression model was used to determine the best weighting coefficient among prognosis-related autophagy genes. The GSE136337 and GSE57317 datasets were used for validation of the model.

Subjects were divided into high‐ and low‐risk cohorts, according to the median ARS in each dataset. The prognostic accuracy of the model was quantified by time-dependent receiver operating characteristic (ROC) curves and calculation of the area under the curve (AUC). Kaplan–Meier curves were generated and analyzed by log-rank test. Other clinical co-variates associated with survival were also analyzed in the training dataset (GSE24080) and the validation dataset (GSE136337) by uni-variable and multi-variable Cox regression analyses.

**Construction and verification of nomogram**

A nomogram combining ARS and ISS was constructed in the training dataset (GSE24080). The calibration curve was used to prove the accuracy and specificity of the nomogram. Time-dependent AUC was used to compare the nomogram and ISS.

**Enrichment analysis**

To identify potential biological pathways differing between the high- and low autophagy risk groups, Gene Ontology analysis was used to conduct on the identified autophagy genes (adjusted p < 0.05) [24]. Higher Gene Ontology scores indicated that autophagy genes were more important, and candidate biological functions and pathways were explored based on functional enrichment analyses [25]. Gene cloud biotechnology information, including the GENEMANIA database (https://genemania.org), the Human Protein ATLAS (https://www.proteinatlas.org), and cytoscape 3.7.2, were used to explore the interactions between model autophagy-related proteins and other known associated proteins.

**Patient selection**

Bone marrow biopsy specimens were from patients newly diagnosed MM (NDMM) and controls with normal bone marrow, who received routine initial diagnosis in the Department of Pathology of our hospital. The clinical study was approved by the Human Ethics Committee of Sun Yat-sen University Cancer Center (Number. G2022-154-01). The study was carried out in accordance with the principles of the Helsinki Declaration. Informed consent was waived because of the study’s retrospective nature and the anonymization of individual data.

**Cell culture and viability analysis**

The multiple myeloma cell lines, NCI-H929, MM.1S, RPMI8226 and U266B1, were purchased from the American Type Culture Collection (Manassas, VA, USA). Cell line authentication was performed by short tandem repeat analysis and cells were regularly tested for absence of mycoplasma contamination using the Myco-Blue Mycoplasma Detection Kit (Vazyme, catalog D101-01). All cells were cultured in a 37°C incubator in a humid atmosphere containing 5% CO_2_. Cells were cultured in RPMI-1640 medium containing 10% fetal bovine serum, supplemented with 100 IU mL^-1^ penicillin and 100 mg mL^-1^ streptomycin.

To explore the expression of autophagy-related proteins, MM cells were treated with the ARNT inhibitor GNF351 (1 μm; MedChemExpress, catalog HY-102023), autophagy inhibitor 3-Methyladenine (3-MA, 10 μm; MedChemExpress, catalog HY-19312), or both for 24 h. To assess drug resistance to Bortezomib, MM cells were cultured for 4 days in the presence of GNF351 (1 μm) or PBS as vehicle control. Alternatively, cells were exposed to a bortezomib concentrations series (0.25–16 nmol L^-1^; QILU Pharmaceutical, catalog HY-10227) for 24 h, after which the cell counting kit-8 assay (CCK-8; APExBIO, catalog K1018) was used to determine cell viability, according to the manufacturer's instructions; cell numbers were counted every 24 h. Furthermore, Annexin V-APC and PI apoptosis kit (KeyGEN, catalog KGA1030-100) was used to detect the apoptosis rate of MM cells treated with vehicle, GNF351, 3-MA or both drugs, respectively.

**Real-time quantitative PCR (RT-qPCR)**

For relative quantification of mRNA expression, total RNA was extracted from primary plasma cells isolated from MM patients and MM cell lines, cDNA was produced by reverse transcription from 1 μg RNA by using Fast Reverse Transcription kit (Esscience, catalog RT001). qPCR was performed using Bio-Rad fluorescent real-time quantitative PCR system (equipment model: LightCycler 480II, USA). The primer set includes GAPDH, ARNT, ATG4D, BIRC5, BNIP3L, CDKN1A, EIF2S1, IRGM, ITGA3, NCKAP1, NRG1, TM9SF1 were purchased from RuiBioTech. The specified genes were amplified according to the kit instructions, and 2^-ΔΔCT analysis was adopted to calculate gene expression. The sequence of above primers was listed in **Table S2**.

**Immunoblot analysis**

Bone marrow samples were lysed, washed with ice-cold PBS, and the cells collected in RIPA lysis buffer containing phenylmethylsulphonyl fluoride and phosphatase inhibitors (Beyotime, catalog P0013B). Total protein concentration was measured using the Pierce BCA Protein Assay Kit (Thermo Fisher Scientific, catalog 23225). Proteins were separated by SDS-polyacrylamide gel electrophoresis, transferred to polyvinylidene difluoride membranes, and probed with primary antibodies against human ARNT (0.25 mg ml^-1^, rabbit monoclonal antibody; Cell Signaling Technology, catalog 5537), microtubule-associated-proteinlight-chain-3 (LC3 I/II; 0.25 mg ml^-1^, rabbit monoclonal antibody; Cell Signaling Technology, catalog 4108), cyclic AMP-dependent transcription factor 3 (ATF3; 0.25 mg ml^-1^, mouse monoclonal; Cell Signaling Technology, catalog MA5-31635), SQSTM1/p62 (sequestosome 1; 0.25 mg ml^-1^, rabbit polyclonal; Cell Signaling Technology, catalog 5114), AKT (0.25 mg ml^-1^, rabbit polyclonal; Cell Signaling Technology, catalog 9272), and β-actin (0.25 mg ml^-1^, rabbit polyclonal; Cell Signaling Technology, catalog 4967). The intensities of protein bands were quantified using Image J software (National Institute of Health, Bethesda, MD, USA) [26].

**Immunohistochemistry (IHC) analysis**

Paraffin-embedded bone marrow biopsy sections (thickness, 5 μm) were prepared. For immunocytochemistry, sections were sequentially incubated with primary anti-ARNT antibody (50 μg ml^−1^, rabbit monoclonal antibody; Cell Signaling Technology, catalog 5537) overnight and anti-rabbit secondary antibody (10 μg ml^−1^; Cell Signaling Technology, catalog 7077) for 1 h at 23°C, followed by development with 3, 30-diaminobenzidine solution (Sigma-Aldrich, catalog D8001-1g). Nuclei were counterstained with hematoxylin (Sigma-Aldrich, catalog H9627-25G). The intensities of positively stained cells were quantified in each of five randomly selected fields using ImageJ software by two independent investigators, blinded to sample identity. For fluorescent staining, sections were incubated with primary antibody and appropriate Alexa Fluor 488-labeled secondary antibodies (Invitrogen, catalog A27034), and sections visualized under a fluorescence microscope (QImaging, Olympus IX71, USA), after counterstaining with DAPI.

**Overexpression and knockdown of ARNT**

To further verify that ARNT may reduce the sensitivity of bortezomib through the autophagy pathway. The ARNT overexpression plasmid (pLVX-EF1a-mNeonGreen-Puro-CMV-hARNT-3Flag) was purchased from Shanghai Lianfeng/Guangzhou Youming Biotechnology Co., Ltd. ARNT shRNA fragments were designed and inserted into the vector pLKD-U6-MCS-CMV-shARNT-EGFP-Puro to construct an ARNT knockdown plasmid. The sequence of above primers was listed in **Table S3**. After packaging the virus in HEK293T cells, NCI-H929 cells were infected.

***In vivo* validated study**

NCI-H929 cells with vehicle or overexpression of ARNT (2 × 10^6^ cells) were injected subcutaneously into the dorsal sides of 4-6-week-old male humanized NRG-3GS mice (NOD.Cg-Rag1^tm1Mom^ Il2rg^tm1Wjl^ Tg(CMV-IL3,CSF2,KITLG)1Eav/J; Jackson Lab, catalog 024099). After 14 days of subcutaneous injection, tumor growth was monitored every 3 days using calipers. After 26 days mice were sacrificed, and tumor volume was measured. The animal study was approved by the Institutional Animal Care and Use Committee of Sun Yat-sen University (Number. L025501202206012).

**Statistical Analysis**

Time-dependent ROC curves were drawn to assess the predictive performance of the autophagy signature in the three cohorts. The AUC was calculated using the survival ROC package. Confidence intervals were calculated using the bootstrap method. Overall survival was defined as the primary outcome and calculated as the time from diagnosis or study entry to death from any cause. Kaplan–Meier curves were drawn using the “survival” package and compared using the log-rank test. Clinical and genetic information were explored for prognostic performance by uni-variable and multivariable Cox analyses, and variables with P value <0.05 were included in the multivariate analysis. A 2-tailed T test or Fisher’s exact test was performed to compare categorical variables. A nomogram was used to visualize and integrate the ARS and ISS for overall survival, the consistency of which was assessed by calibration. AUC values were used to evaluate and compare the prognostic value of candidate factors. All statistical analyses were performed using R software (version 3.6.0) and SPSS version 24.0 software (SPSS, Inc., Chicago, IL, USA). A two-sided P < 0.05 was considered statistically significant.
