# Supplementary material for: A new autophagy-related nomogram and mechanism in multiple myeloma
Source: Genes Dis. 2023 Sep 21;11(5):101120. doi: 10.1016/j.gendis.2023.101120 (PMC11145194; doi:10.1016/j.gendis.2023.101120)
Supplement: Multimedia component 2 [file mmc2.docx]

**Supplementary Materials**

**Table S1. Eleven autophagic genes selected by multi-COX regression analysis to establish a new prognosis-related autophagy risk score (ARS).**

| **Gene** | **Coefficient** | **HR** | **HR.95L** | **HR.95H** | ***P* value** |
| --- | --- | --- | --- | --- | --- |
| ARNT | 2.12832732 | 8.40080318 | 1.31374809 | 53.7191984 | 0.02456215 |
| ATG4D | -2.43411590 | 0.08767523 | 0.01356265 | 0.56677328 | 0.01058072 |
| BIRC5 | 1.40225999 | 4.06437505 | 1.42595234 | 11.5846400 | 0.00869163 |
| BNIP3L | -2.48077110 | 0.08367868 | 0.01388913 | 0.50414398 | 0.00678054 |
| CDKN1A | -2.59942260 | 0.07431648 | 0.01938776 | 0.28486727 | 0.00014966 |
| EIF2S1 | 2.47848390 | 11.9231740 | 0.92962762 | 152.923682 | 0.05692199 |
| IRGM | -0.49249200 | 0.61110160 | 0.33533828 | 1.11363715 | 0.10773733 |
| ITGA3 | -0.91072830 | 0.40223118 | 0.13286520 | 1.21769975 | 0.10708079 |
| NCKAP1 | -0.78227780 | 0.45736303 | 0.20565023 | 1.01716853 | 0.05508282 |
| NRG1 | -1.52397470 | 0.21784429 | 0.04407439 | 1.07672802 | 0.06158299 |
| TM9SF1 | -3.19568560 | 0.04093845 | 0.00467746 | 0.35830495 | 0.00388583 |

**Table S2. The sequence of primers used in this study.**

| **Primers Name** | **Sequence** |
| --- | --- |
| GAPDH-F | TGTTGCCATCAATGACCCCTT |
| GAPDH-R | CTCCACGACGTACTCAGCG |
| ARNT-F | GAATTGGACATGGTACCAGG |
| ARNT-R | AAGCTGATGGCTGGACAATG |
| ATG4D-F | TATGGGCCATCGCTAGTGG |
| ATG4D-R | TCTGAGAGTTGGGGCACATAC |
| BIRC5-F | AGGACCACCGCATCTCTACAT |
| BIRC5-R | AAGTCTGGCTCGTTCTCAGTG |
| BNIP3L-F | CAATGTCGTCCCACCTAGTCG |
| BNIP3L-R | TAGCTCCACCCAGGAACTGTTG |
| CDKN1A-F | GGAAGGGACACACAAGAAGAAG |
| CDKN1A-R | AGCCTCTACTGCCACCATCTTA |
| EIF2S1-F | AGCCCTAAGAGCAGGTTTGA |
| EIF2S1-R | GCCTTCTGTTCTCTCCAGG |
| IRGM-F | GCTTGAAAAAGAGCAGAGCATT |
| IRGM-R | GGGCCCAACTGAAGTGAG |
| ITGA3-F | TCAACCTGGATACCCGATTCC |
| ITGA3-R | GCTCTGTCTGCCGATGGAG |
| NCKAP1-F | AGTGTACCCTTAGTGACCAGTTGCT |
| NCKAP1-R | TCAGGTTCCCCTTTCTTACCAGT |
| NRG1-F | CCCGCCGGCTATTGGTGACTT |
| NRG1-R | ATGACCACCCCGGCTCGTATGT |
| TM9SF1-F | ATGACCTGGCTCGGTACAAC |
| TM9SF1-R | TGTCACCCTGGTCAAAGTCA |

**Table S3. The shRNA sequence of ARNT.**

| **Name** | **Sequence** |
| --- | --- |
| H-sh1-ARNT-F-AgeI | CCGGGAGAAGTCAGATGGTTTATTTCTCGAGAAATAAACCATCTGACTTCTCTTTTTG |
| H-sh1-ARNT-R-EcoRI | AATTCAAAAAGAGAAGTCAGATGGTTTATTTCTCGAGAAATAAACCATCTGACTTCTC |

**Figure Legends**

**Figure S1. Supplement to the autophagy model.**

**(A)** Workflow of prognostic autophagic gene signatures. **(B)** Autophagy-related genes (n = 38) associated with differences in prognosis of patients with MM (*P* < 0.02). HR > 1, high risk gene; HR < 1, low risk gene. **(C)** The formula of the ARS model. **(D-E)** Time-dependent ROC analysis of prognostic model prediction of 1-, 3-, and 5-year overall survival in two validation datasets. **(F-G)** Kaplan–Meier curve of the prognostic model in two validation datasets. **(H-I)** Forest plot of univariate (green) and multivariate (red) Cox regression analysis in the GSE136337 validation dataset. Multi-factor inclusion criteria: single factor p<0.05, and reproducible factors were excluded. Abbreviations: MM: multiple myeloma; HR: hazard ratio; ARS: autophagy risk rcore; ROC: receiver operating characteristic.

**Figure S2. ARNT is closely related to autophagy, immunity and endoplasmic reticulum stress.**

**(A)** ARNT and AKT correlation network analyzed using the GENEMANIA database. Correlations are illustrated by different colored lines. **(B)** Scatter plot showing the intensity of ARNT expression in PBMC. All cells color scale - % of max. **(C)** ARNT expression in various immune cells from PBMC. **(D)** Heatmap showing the correlation of ARNT with different cell type markers. **(E)** IHC staining showing the expression of ARNT and ER stress in different cell lines. Data was obtained from The Human Protein ATLAS (<https://www.proteinatlas.org/humanproteome/tissue>). Abbreviations: PBMC: peripheral blood mononuclear cell; IHC: immunohistochemistry; ER: endoplasmic reticulum.
