# Supplementary material for: A new autophagy-related nomogram and mechanism in multiple myeloma
Source: Genes Dis. 2023 Sep 21;11(5):101120. doi: 10.1016/j.gendis.2023.101120 (PMC11145194; doi:10.1016/j.gendis.2023.101120)

Supplementary Figure 1

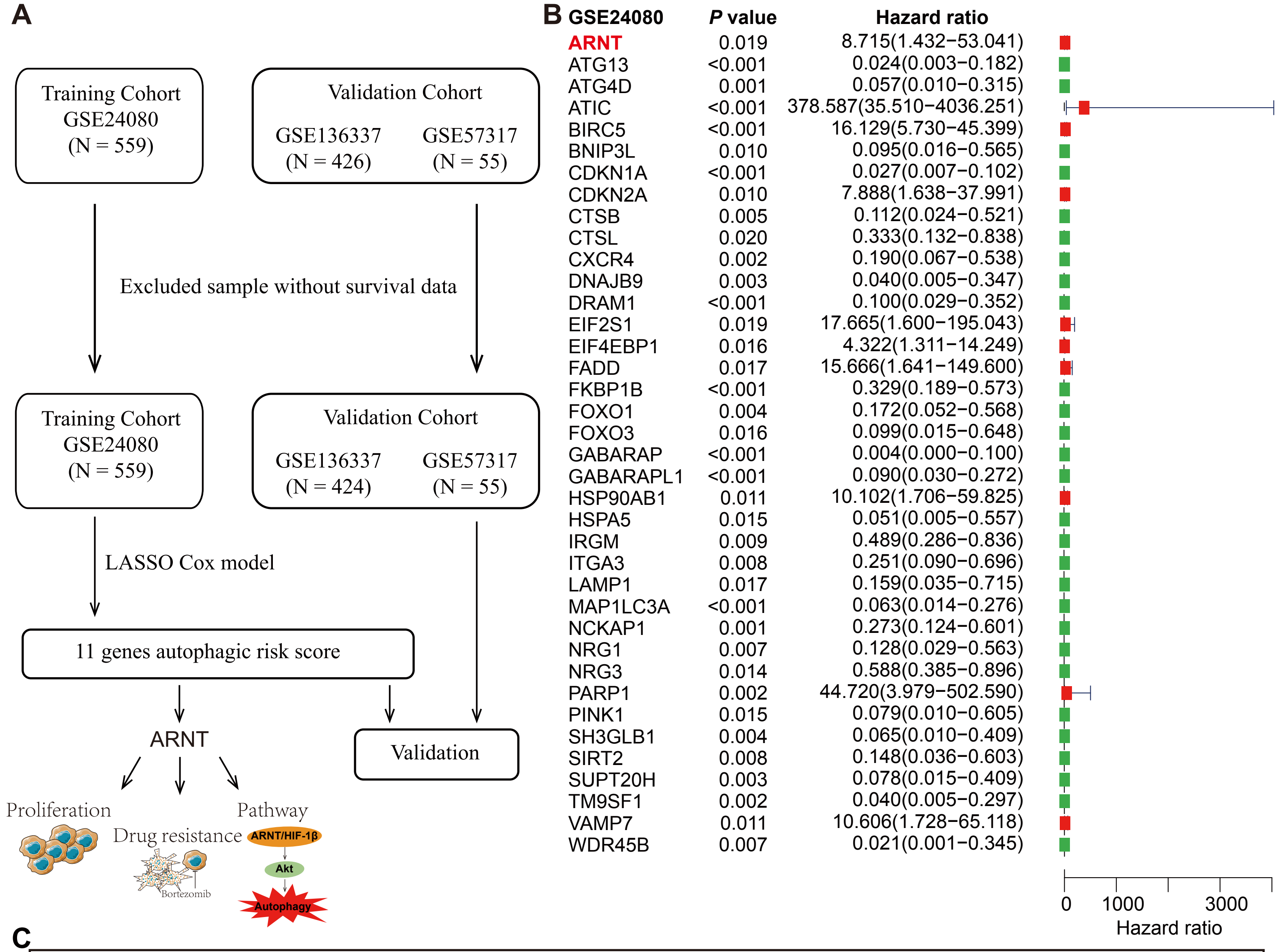

C

ARS = (2.13 × expression of ARNT) + (2.48 × expression of EIF2S1) + (1.40 × expression of BIRC5) – (0.49 × expression of IRGM) – (0.78 × expression of NCKAP1) – (0.91 × expression of ITGA3) – (1.52 × expression of NRG1) – (2.43 × expression of ATG4D) – (2.48 × expression of BNIP3L) – (2.60 × expression of CDKN1A) – (3.20 × expression of TM9SF1).

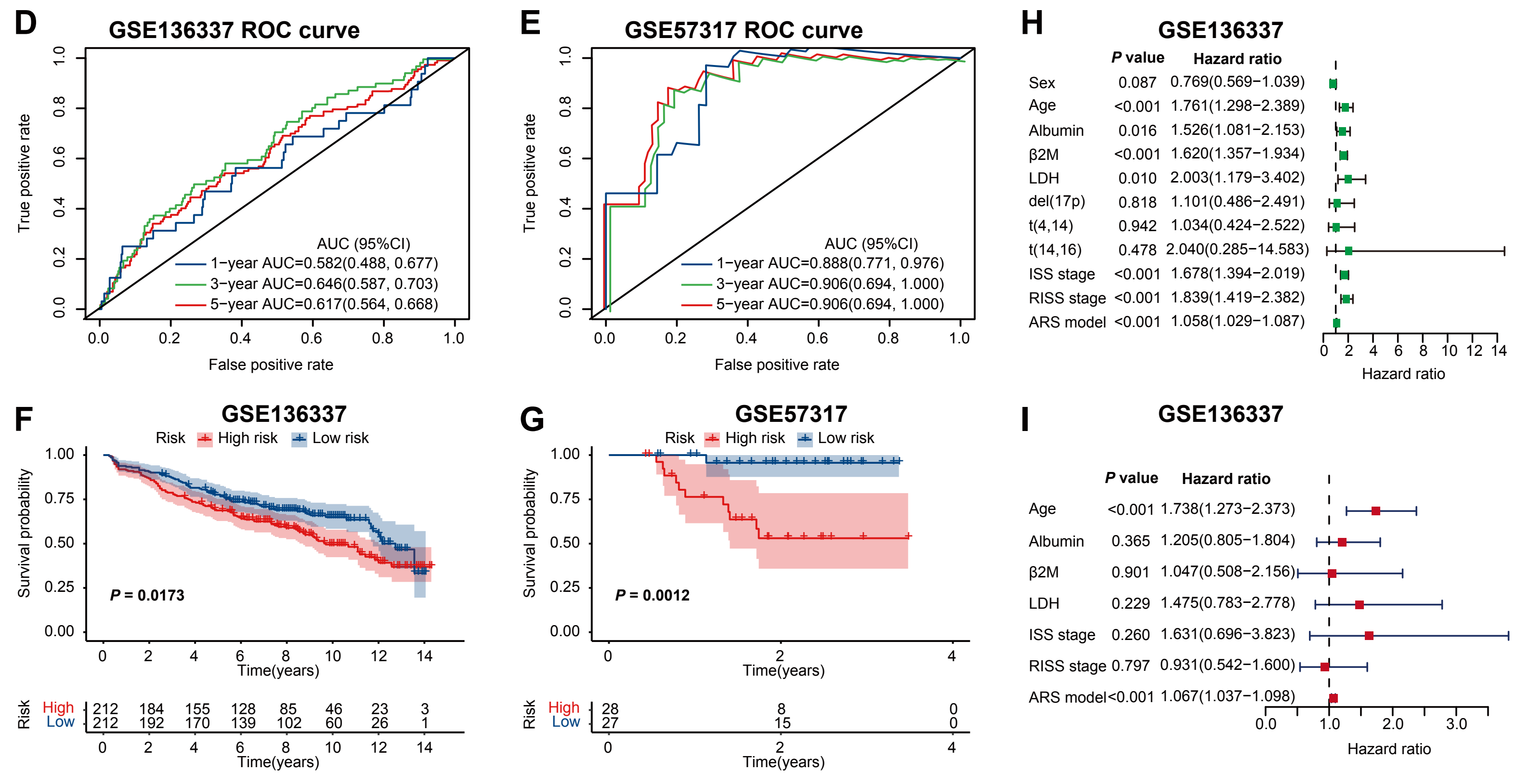

Supplement: Multimedia component 3 [file mmc3.pdf]
