# Supplementary figures and images for: A new autophagy-related nomogram and mechanism in multiple myeloma
Source: Genes Dis. 2023 Sep 21;11(5):101120. doi: 10.1016/j.gendis.2023.101120 (PMC11145194; doi:10.1016/j.gendis.2023.101120)

Supplementary Figure 2

A

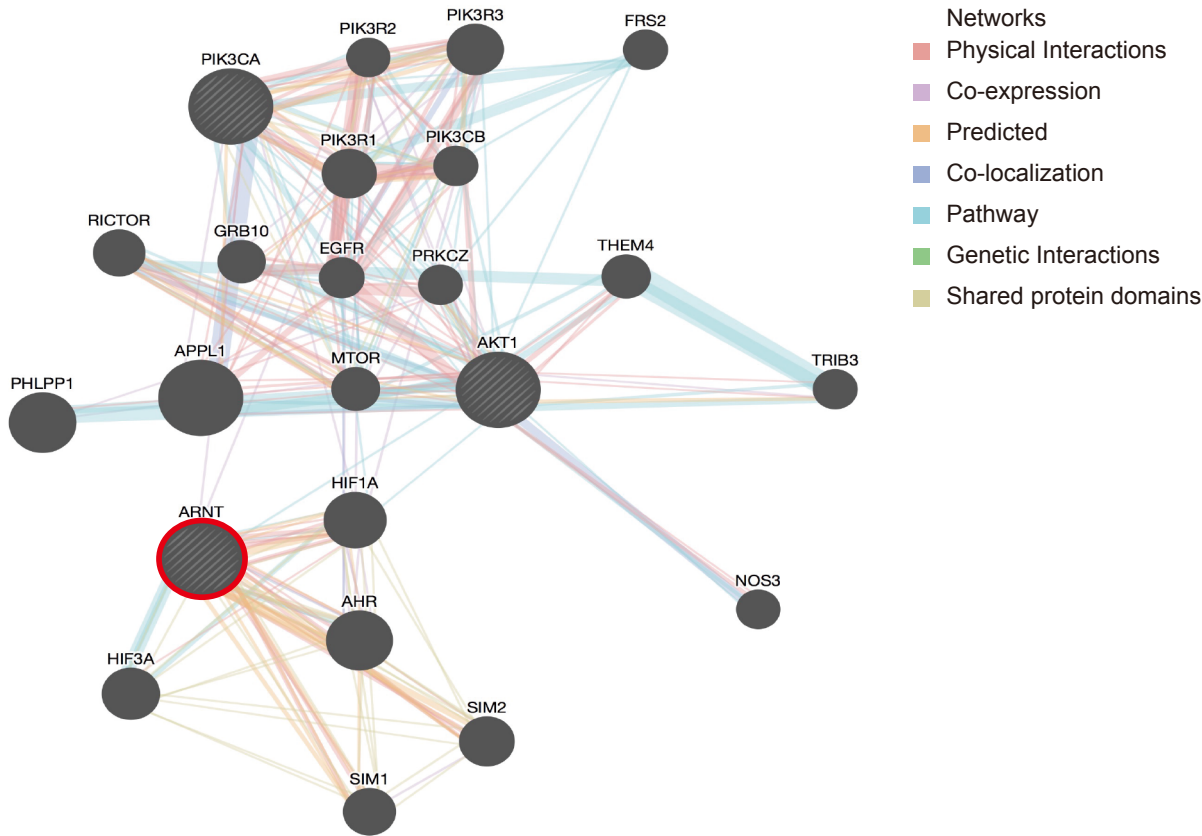

B

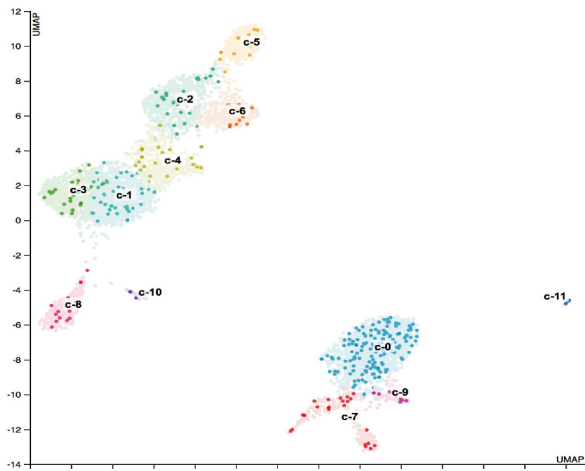

C

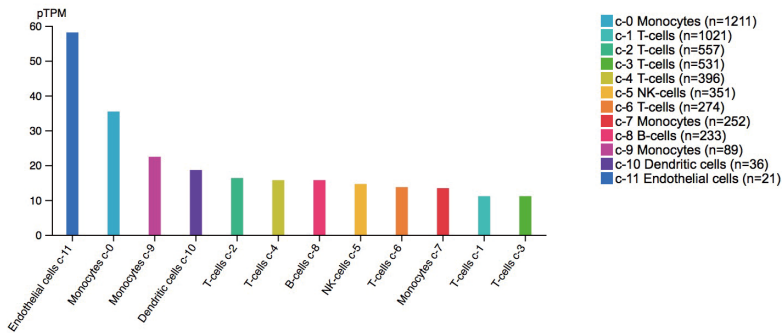

D

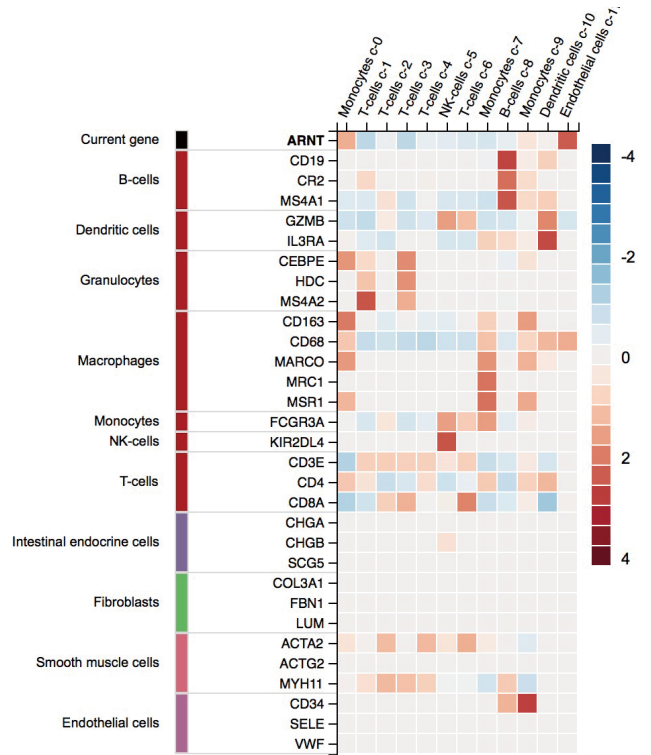

E

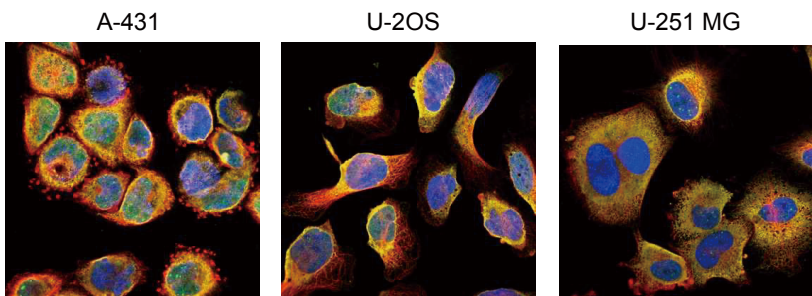

Supplement: Multimedia component 4 [file mmc4.pdf]
